# Selective activation of central thalamic fiber pathway facilitates behavioral performance in healthy non-human primates

**DOI:** 10.1038/s41598-021-02270-7

**Published:** 2021-11-29

**Authors:** A. P. Janson, J. L. Baker, I. Sani, K. P. Purpura, N. D. Schiff, C. R. Butson

**Affiliations:** 1 Department of Biomedical Engineering, University of Utah, Salt Lake City, UT USA; 2 Scientific Computing and Imaging Institute, Salt Lake City, UT USA; 3 Departments of Neurology and Neurosurgery, Vanderbilt University Medical Center, Nashville, TN USA; 4 Feil Family Brain and Mind Research Institute, Weill Cornell Medicine, New York, NY USA; 5 The Rockefeller University, New York, NY USA; 6 Department of Basic Neurosciences, University of Geneva, Geneva, Switzerland; 7 Departments of Neurology, Neurosurgery, and Psychiatry, Salt Lake City, UT USA; 8 Norman Fixel Institute for Neurological Diseases, University of Florida, Gainesville, FL USA

**Keywords:** Neuroscience, Cognitive neuroscience, Neural circuits

## Abstract

Central thalamic deep brain stimulation (CT-DBS) is an investigational therapy to treat enduring cognitive dysfunctions in structurally brain injured (SBI) patients. However, the mechanisms of CT-DBS that promote restoration of cognitive functions are unknown, and the heterogeneous etiology and recovery profiles of SBI patients contribute to variable outcomes when using conventional DBS strategies,which may result in off-target effects due to activation of multiple pathways. To disambiguate the effects of stimulation of two adjacent thalamic pathways, we modeled and experimentally compared conventional and novel ‘field-shaping’ methods of CT-DBS within the central thalamus of healthy non-human primates (NHP) as they performed visuomotor tasks. We show that selective activation of the medial dorsal thalamic tegmental tract (DTTm), but not of the adjacent centromedian-parafascicularis (CM-Pf) pathway, results in robust behavioral facilitation. Our predictive modeling approach in healthy NHPs directly informs ongoing and future clinical investigations of conventional and novel methods of CT-DBS for treating cognitive dysfunctions in SBI patients, for whom no therapy currently exists.

## Introduction

The central thalamus (CT) is a key node in the arousal regulation network of the mammalian brain hypothesized to modulate large-scale activity patterns across the anterior forebrain in response to internal and external demands during wakefulness^1^. Damage of the CT in humans, due to traumatic brain injury (TBI) or stroke, results in enduring cognitive deficits in the allocation of attention, maintenance of concentration and focus, working memory, impulse control, processing speed, and motivation^2–6^. As current therapeutics are not effective at treating these cognitive deficits, deep brain stimulation (DBS) within the central thalamus (CT-DBS) has been proposed as a therapeutic option to artificially restore arousal regulation in order to reestablish and/or broadly support cognitive function in SBI patients^7–9^. A single case study from our group^10^ demonstrated that by targeting the ‘wing’ of the central lateral (CL) nucleus^11–13^, the use of CT-DBS can result in a significant and cumulative improvement in a patient’s responsiveness, communication, and motor function following a very severe TBI. However, the mechanisms that produced this outcome, which are dependent on DBS lead location and methods of neural activation, remain unknown. The use of DBS to treat very severe TBI patients has a long history of failure, primarily due to poor patient selection and hypothesis-free DBS targeting (reviewed in^14^). The predominant target for DBS in these patients has been the centromedian-parafascicularis complex (Cm-Pf) of the thalamus^15–18^, a relatively large and prominent nucleus adjacent to the CL nucleus. Yet to date, clinical outcomes in this patient population have been highly variable due to several factors such as the etiology of patients investigated, the ability to successfully target and acquire CM-Pf during lead implantation, and the background spontaneous recovery rate from SBIs within the first year following an injury^19^.

Despite the variability in clinical results, the preclinical evidence for enhancing arousal and behavioral performance in intact animals during electrical stimulation of CL is more extensive^20^. Recent studies confirm that electrical stimulation of CL can effectively enhance arousal^21^ and performance^22–26^ in healthy rodents and in two rodent models of pathology, epilepsy^27–30^ and TBI^31^. In anesthetized animals, optogenetic stimulation of CL in mice^32^ and electrical stimulation of CL in rodents^26^ and non-human primates (NHP)^33,34^ demonstrate broad cortical and subcortical activations. A recent study in healthy behaving non-human primates (NHPs) expands on these results by examining the effects of various methods of CT-DBS on behavior and physiology while the animals performed complex visuomotor tasks^35^. A unique aspect of this study^35^ was the use of two closely spaced DBS leads placed within CT and the discovery that both the precise location of the leads in CT and the orientation of the electric field established between the two leads were critical parameters for improving performance and enhancing frontostriatal activity patterns. This discovery led us to hypothesize that the key target for stimulation is the local fiber tracts that traverse the CT and not a single nucleus. One candidate pathway is the medial dorsal tegmental tract (DTTm), a component of the ascending reticular activating system that passes through CL and the thalamic reticular nucleus (TRN) broadly into the cortex and striatum^36^. The DTTm also carries glutamatergic efferents from the CL nucleus to the TRN, cortex, and striatum^37^. Another candidate fiber pathway is the glutamatergic fibers emanating from Cm-Pf that project, through the TRN enroute, predominantly to the striatum^38–42^. Our focus in this study was to examine how various methods of CT-DBS recruit these two intra-thalamic pathways and how their selective or combined activation may influence performance in the healthy NHP.

This a priori comparison between the two pathways was chosen for two reasons: (1) in vivo studies demonstrate that these intra-thalamic pathways reciprocally inhibit the overall central (or ‘intralaminar’) thalamic nuclei^43,44^, and (2) human DBS studies suggest that behavioral facilitation may be achieved with either pathway’s activation^10,15,16^. Thus, the null hypothesis for electrical activation of the central thalamus more generally maintains that bulk activation of the two pathways might be synergistic^45^. Here, we partially falsify this null hypothesis and show that selective activation of the DTTm produces behavioral facilitation in the healthy NHP.

A precise therapeutic DBS target may be difficult to determine for many SBI patients given the presence of a wide range of structural injuries in this population including substantial deformation and atrophy of the thalamic nuclei^46^. However, patients with higher levels of consciousness and less structural injury of their thalamus, frontal lobe, and striatum are expected to be ideal candidates for DBS therapy as they often suffer from enduring cognitive dysfunction^8^. In such persons, however, improved targeting and activation of the arousal related pathways that minimizes OFF-target side effects, are critical to developing this potential therapy, as recently demonstrated^47^. The study presented here further establishes the DTTm fiber pathway as an optimal DBS target to facilitate performance in healthy NHPs and directly informs ongoing^47^ and future clinical studies using DBS to treat the enduring fatigue and cognitive dysfunction experienced by the majority TBI patients.

## Results

### Targeting of central thalamic arousal regulation pathways for deep brain stimulation

**Figure 1:**
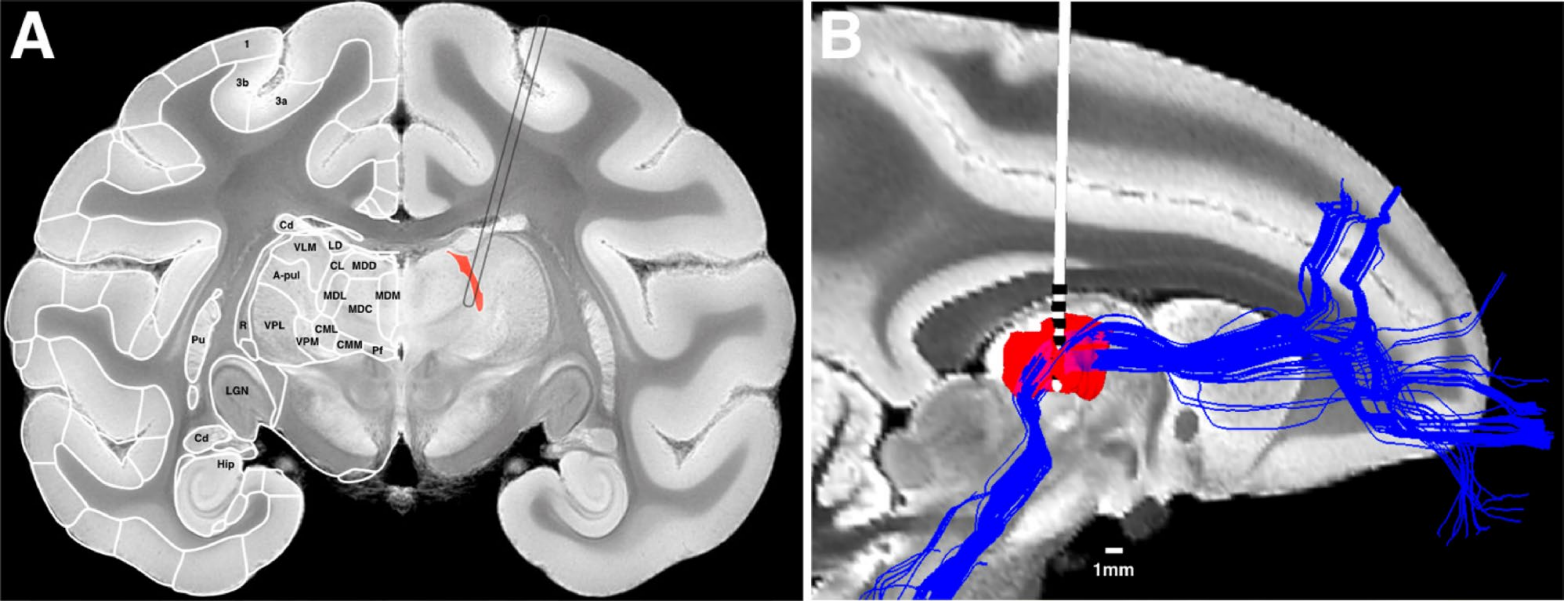
Targeting the central lateral (CL) nucleus and DTTm fiber pathway in healthy NHPs with scaled multi-contact DBS leads. (**A**) Coronal view of a gradient echo (GRE) image from the NHP MRI-DTI brain atlas used in this study (Calabrese et al., 2015) with cortical across consecutive trials^53^ hemisphere. CL is colored in red in the right hemisphere and the schematic outline of the scaled DBS lead (0.84 mm OD) illustrates the trajectory used to target CL and the DTTm fiber pathway. Shown is image 83 (148), − 9.3 mm from the anterior commissure, which can be found here: https://scalablebrainatlas.incf.org/macaque/CBCetal15. APUL—anterior pulvinar; CL—centrolateral thalamic nucleus; Cm-Pf—centromedian thalamic nucleus, lateral part; CM—centromedian thalamic nucleus, medial part; Hip—hippocampus; LGN—lateral geniculate nucleus; MDC—mediodorsal thalamic nucleus, central part; MDD—mediodorsal thalamic nucleus, dorsal part; MDL—mediodorsal thalamic nucleus, lateral part; MDM—mediodorsal thalamic nucleus, medial part; Pf—parafascicularus nucleus; Pu—putamen; R—reticular thalamic nucleus; VLM—ventral lateral thalamic nucleus, medial part; VPL—ventral posterolateral thalamic nucleus; VPM—ventral posteromedial thalamic nucleus; 1—area 1 of cortex (somatosensory); 3a—area 3a of cortex (somatosensory); 3b—area 3b of cortex (somatosensory). **(B)** Sagittal view of the T2 imaging used for surgical planning of DBS lead trajectories. Here one of the three six contact DBS leads (NuMed Inc.) implanted into the right thalamus of NHP3 is shown. The CL nucleus, shown in red, and the targeted fibers of the DTTm reconstructed from a high-resolution ex vivo dataset (see “Methods” section, Sani et al., 2019), shown in blue, illustrate the pathway of brainstem and anterior forebrain projections of fibers passing through and/or originating in the CL nucleus. The geometry of the CL nucleus spans ~ 5 mm A-P, 4 D-V, 1 mm M-L in the adult NHP.

Our goal in this study was to target the central lateral (CL) nucleus of the NHP thalamus (Fig. 1A) with multiple scaled DBS leads^48^ for long-term behavioral experimentation. This study uses previously collected behavioral and imaging data (NHP1 and NHP2) and new behavioral and imaging data collected in a third animal (NHP3) to construct a new biophysical modeling framework^49^ to investigate differential activation of derived fiber pathways within the NHP thalamus. Previously we had shown that several specific CT-DBS anode–cathode configurations resulted in consistent facilitation, while others showed suppression or no effect on behavioral performance. The facilitatory configurations also generally followed an inverted-U relationship with the amplitude of stimulation^35^. We hypothesized from these results that behavioral facilitation was likely a result of bulk activation of the medial dorsal tegmental tract (DTTm)^36^. Therefore, we preoperatively positioned multiple DBS leads into the NHP thalamus of an additional animal in order to maximally cover the anterior to posterior expanse of CL and the DTTm fiber pathway (as shown in blue in Fig. 1B). Utilizing our new biophysical modeling framework^49^ we specifically modeled the selective fiber activation within the two distinct pathways in all three animals. The DTTm was modeled using deterministic tractography (see “Methods” section) by first seeding the brainstem pedunculopontine nucleus (PPN) and then constraining fibers that passed through the CL and TRN nuclei, which then projected to prefrontal areas (as seen in Fig. 1B). Upon entering the ventral region of the thalamus from the brainstem, the DTTm projects rostrally across the span of the CL nucleus, through the dorsal head of the TRN, and into the internal commissures before radiating outward to prefrontal areas (see Supplementary Fig. 1 for additional views). Detailed segmentations of CL, Cm-Pf, and the TRN were taken from a full-brain atlas of the macaque monkey^50^ and registered to each animal’s model.

**Figure 2:**
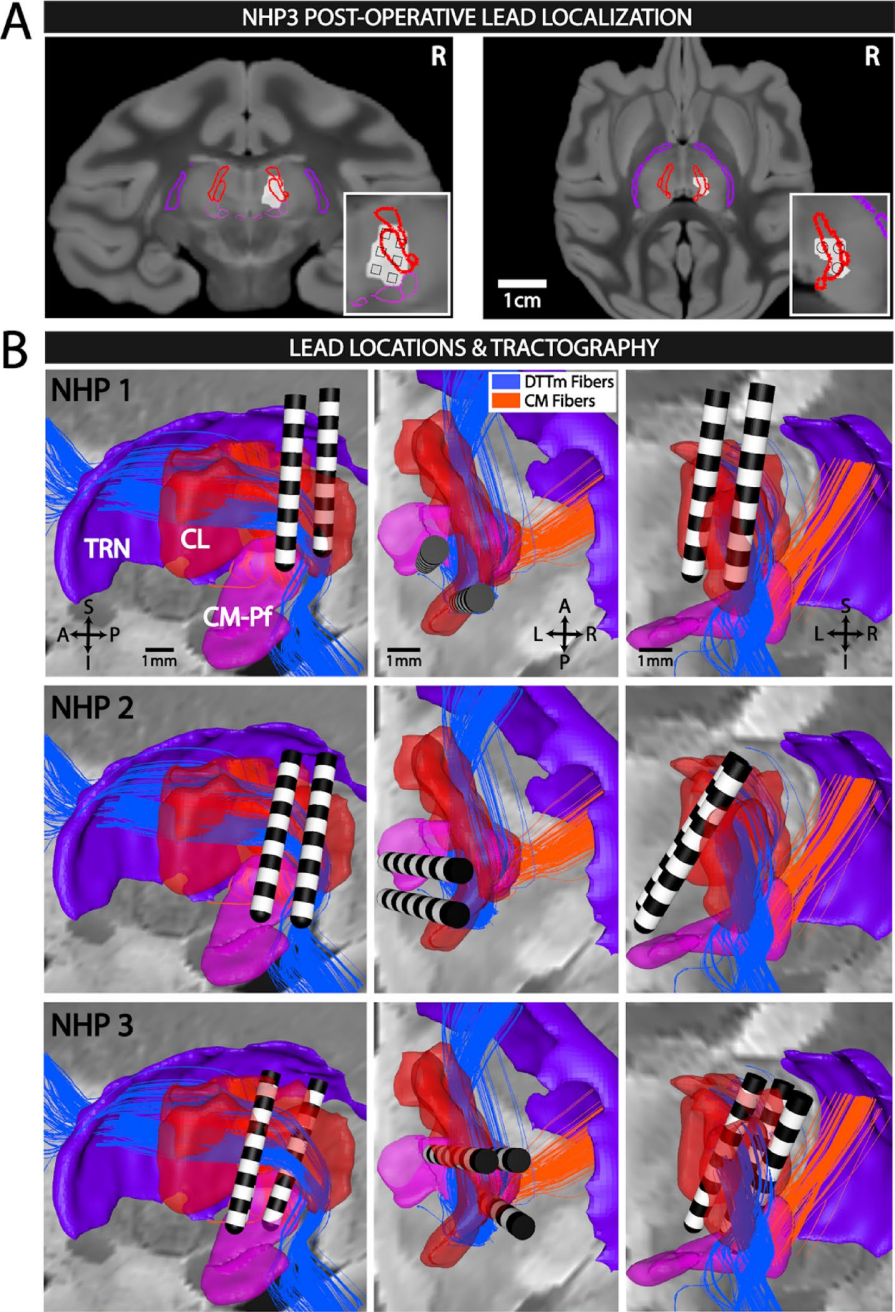
Central thalamic deep brain stimulation (CT-DBS) of primary ascending arousal system pathway. (**A**) Post-operative localization of the three DBS leads in the right thalamus of NHP3, with axial (left) and coronal (right) views. The computed tomography artifact of the DBS lead contacts, see insets with individual contact, is fused with the macaque MRI atlas and overlaid with segmentations of the thalamic reticular nucleus (TRN) shown in purple, the central lateral (CL) nucleus in red, and the centromedian-parafascicularus complex (Cm-Pf) in magenta. (**B**) Reconstruction of the lead locations for the three NHP subjects in the sagittal (left), axial (middle), and coronal (right) planes, along with the thalamic nuclei in A and the two predominant fiber pathways: the medial Dorsal Tegmental Tract (DTTm) shown in blue and the Cm-Pf fibers in orange.

In each animal post-implant computed tomography imaging was used to visualize the metallic artifacts produced by the individual DBS lead contacts (Fig. 2A) thereby enabling accurate registration of the virtual DBS leads into each animal’s biophysical model^49^ (see “Methods” section). The result of this registration is detailed in Fig. 2A for NHP3 and the contours of the individual contacts (see insets) can be clearly identified for each of the three DBS leads implanted. Using this method, the reconstructed DBS lead placements for each animal relative to the targeted CL nuclei (shown in red) along with two fiber pathways are shown in Fig. 2B. In addition to the DTTm fiber pathway (blue fibers in Fig. 2B), the Cm-Pf nucleus was used generate the predominant fiber pathway also projecting through TRN (orange fibers in Fig. 2B). As seen in Fig. 2B, the DTTm fibers, specifically modeled and targeted in NHP3, are clearly segregated from the Cm-Pf fibers as they project through the TRN (see Supplementary Fig. 1 for additional views).

### Behavioral performance was influenced by choice of stimulation configuration and amplitude

**Figure 3:**
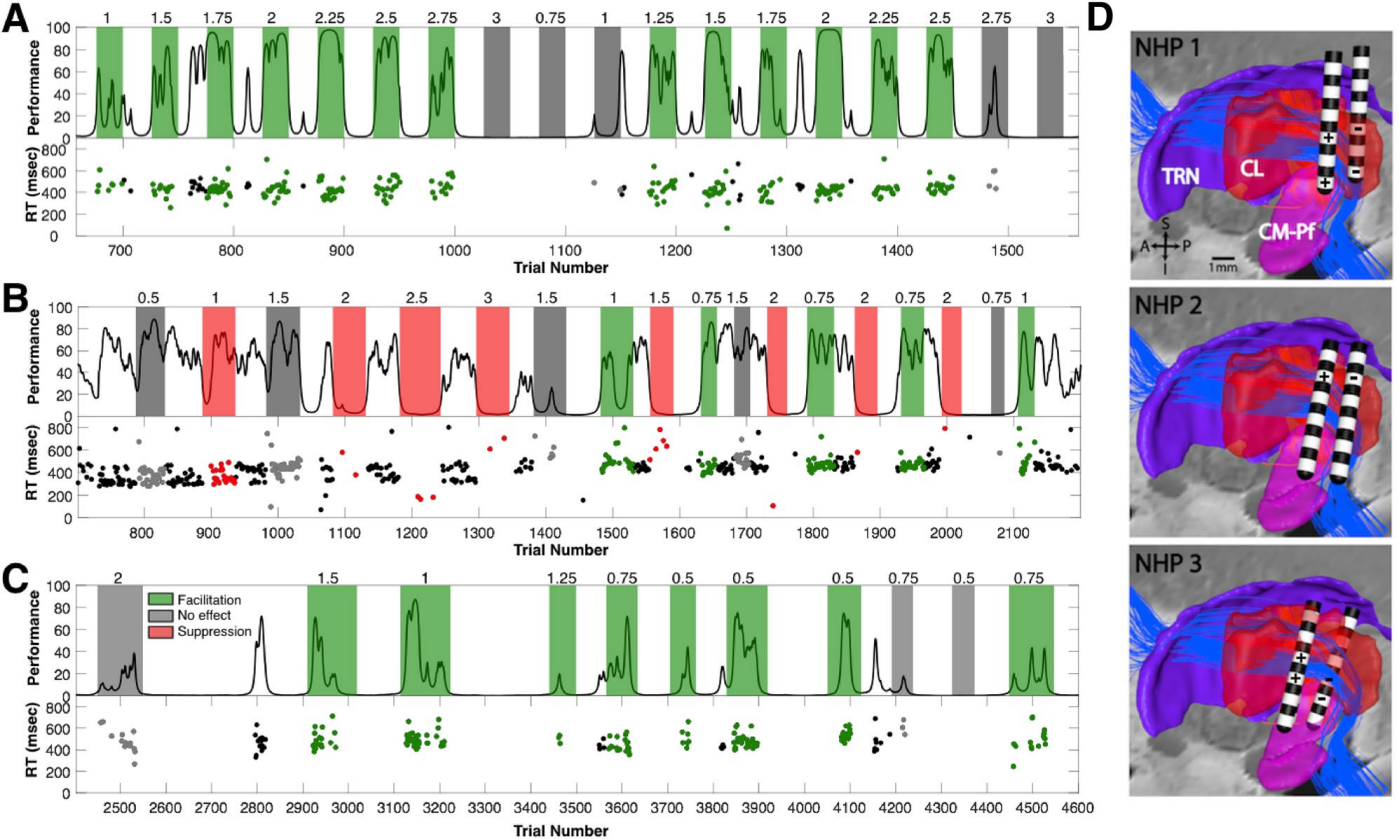
The effects of CT-DBS amplitude on behavioral performance across animals. (**A**) The performance estimate of NHP1 on the visuomotor reaction time task is shown in the upper plot as a smoothly varying black line and thalamic boundaries^51^ shown on the left. Periods of continuous high-frequency CT-DBS are colored according to the significance of the LOR value (*p* < 0.05); facilitation in green, suppression in red, and gray for no effect. Stimulation amplitudes (0.75–3.0 mA) are noted above each CT-DBS period and the same anode–cathode configuration was used. The lower plot shows the reaction times of correctly performed trials with the same color code; reaction times are in black for CT-DBS OFF periods. **(B)** Same as in A, but for NHP2 (*partial reproduction of* Fig. 2C,D in *Baker et. al., 2016*). In this session, CT-DBS stimulation amplitudes greater than 1.5 mA significantly suppressed performance whereas amplitudes 1.5 mA and below had either no effect or modestly facilitated performance. The same anode–cathode configuration was used throughout. **(C)** Same as in A and B, but for NHP3. This animal performed a variation of the vigilance task that required more engagement with the task (see “Methods” section); hence, the great number of trials. **(D)** Reconstruction of the lead locations for each NHP shown in the sagittal plane, along with the CL, CM and TRN nuclei and the fibers of the DTTm are shown in blue. The anode (+) and cathode (−) field-shaping configurations are shown for each.

The most consistent facilitation of behavioral performance across the three NHPs (shown in Fig. 3) was obtained with anode–cathode configurations using the lower three contacts of the two DBS leads in NHP1, the upper two contacts of the two DBS leads in NHP2, and the middle contacts of the caudal and rostral lateral DBS leads in NHP3 (see Fig. 2B). At the start of most experimental sessions the animals typically performed above 75% correct during the first 600+ trials. Performance then typically decreased until the animal was satiated and ceased working^35,52^. Around the time of this natural performance decrement, the use of CT-DBS was able to facilitate and/or the restore performance, depending on the configuration and amplitude of stimulation (Fig. 3). In these three example sessions, anodes were placed on the caudal lead and cathodes were placed on a rostral lead; however, the amplitude of stimulation was a key factor in all three animals (Fig. 3). To quantify the effect of CT-DBS on performance, the log of the odds ratio (LOR) was used to compare performance during trials just prior to CT-DBS onset to the block of trials during CT-DBS (see “Methods” section). In NHP1 (Fig. 3A), current levels between 1.0 and 2.5 mA facilitated performance (green periods, positive LOR values, *p* < 0.05), while current levels below or above this range had no or minimal effect on performance (gray periods, Fig. 3A). In NHP2, current levels from 0.25 to 1 mA had either no effect or facilitated performance (Fig. 3B), while amplitudes 1.5 mA and above consistently suppressed performance (red colored periods, negative LOR, *p* < 0.05). In NHP3 amplitudes 0.5 to 1.5 mA facilitated performance while amplitudes 2 mA and higher tended to suppress performance (Fig. 6, also see Supplementary Fig. 2). In NHP2 and NHP3, stimulation amplitudes above 2 mA tended to reduce or significantly suppressed performance when the effective field-shaping configurations were used (Fig. 3B,C). Of note, amplitudes above 2.5 mA in NHP1 had similar suppressive effects on performance (Fig. 3A). The corresponding anode–cathode configurations used to enhance each animal’s performance are shown in Fig. 3D. We observed that in all three animals, when the shape of the electrical field was parallel to the DTTm fiber pathway (Fig. 3D) and within a range of stimulation amplitudes, performance could be enhanced.

**Figure 4:**
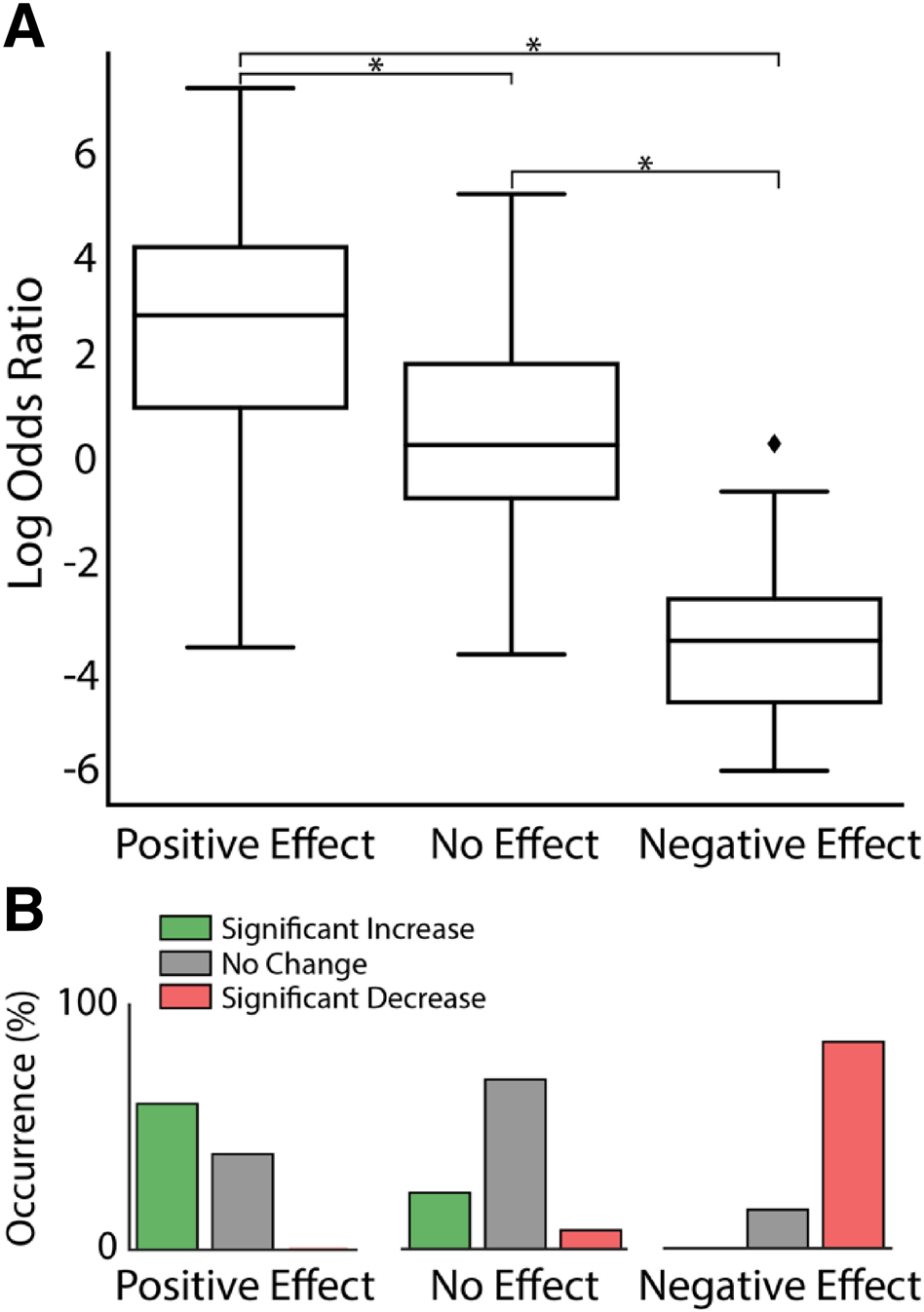
CT-DBS configuration-dependent effects on behavioral performance. (**A**) Stimulation configurations across the three animals are grouped by the configurations that had a positive effect on performance (left), no effect (middle), and a negative effect (right) and box plots showing the distribution of log odds ratio (LOR) changes. Each configuration group has significantly different effects on performance compared to the other two groups, with a two-tailed t-test and all *p*-values < 0.001. (**B**) The normalized occurrence of significant increases, significant decreases, and no significant change for the same three configurations groups determined by the significance of the LOR score at an α level of 0.05. Configurations in the positive effect group predominantly show significant increases in performance with no occurrence of significant decreases whereas configurations in the negative effect group predominantly show the opposite.

To summarize the effects of the anode–cathode configurations that facilitated performance across animals, the LOR for all these DBS periods were grouped into three effect groups, positive effect, no effect, and negative effect (Fig. 4A). Unpaired, two-tailed t-tests show that configurations in the positive effect group significantly increase behavioral performance over the no effect and negative effect groups. The negative effect group significantly decreased behavioral performance over the no effect group, all with a *p*-value < 0.001. Figure 4B illustrates the normalized occurrence of a configuration in that group either facilitated, suppressed, or producing no significant change in performance during stimulation. The occurrence of performance facilitation is highest in the positive effect group, the occurrence of no significant effects is highest in the no effect group, and the occurrence of performance suppression is highest in the negative effect group. The distribution of these effects in each group emphasizes the choice of group for each configuration. Although the labels were manually assigned based upon knowledge of initial experimental results, the significant difference in performance between groups demonstrate that behavioral effect can be determined by configuration alone. These results also demonstrate that the NHPs were not trained to perform only in response to DBS since several configurations (see the no effect group) did not significantly change behavioral performance, even at similar amplitude levels to configurations in the positive effect group. To better understand these amplitude and configuration dependences we used the biophysical model to provide more insight into the specific activation of the DTTm and Cm-Pf fiber pathways.

### Shaping of the electrical field selectively activates the medial dorsal tegmental tract (DTTm) and improves behavioral performance

**Figure 5:**
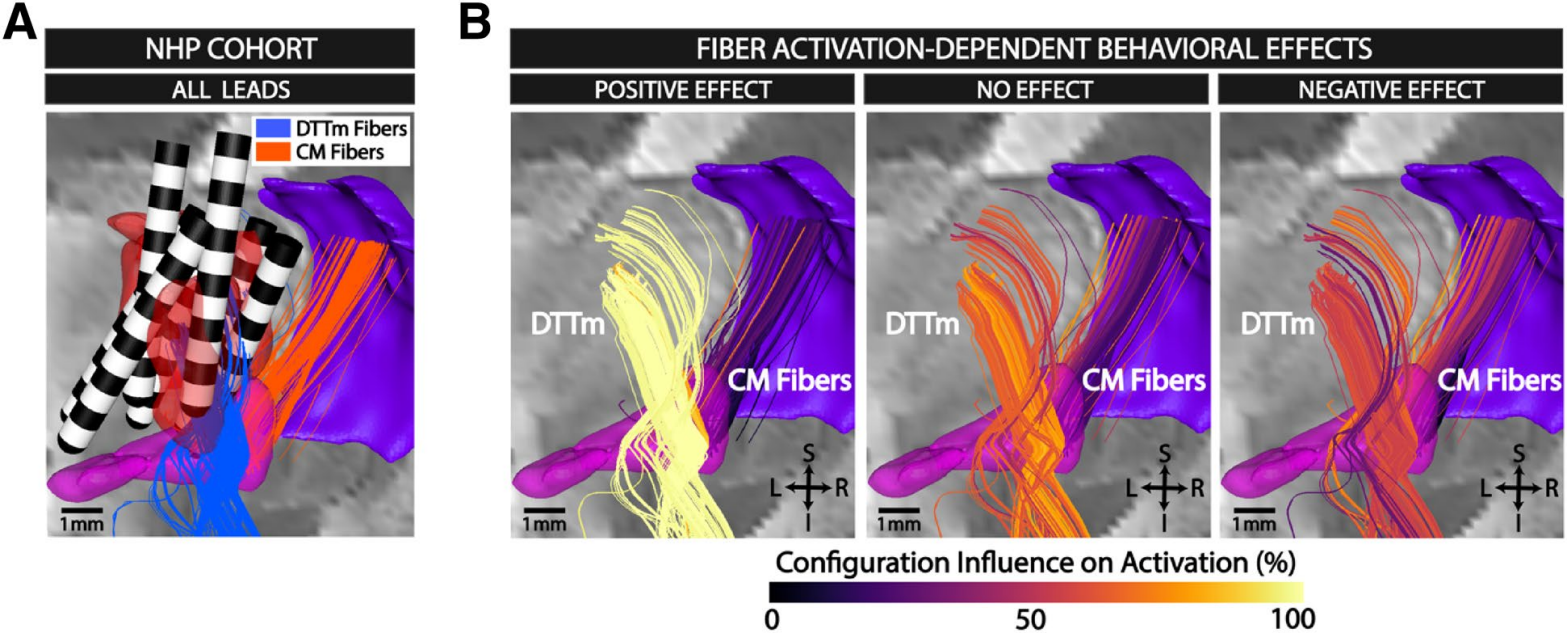
Effective stimulation configurations selectively activate thalamic pathways. (**A**) Combined lead locations of all three NHPs in one coronal image with the two predominant fiber pathways: the DTTm in blue and the Cm-Pf fibers in orange. (**B**) Stimulation configurations across all animals grouped by those that had a positive effect on performance (left), no effect (middle), and a negative effect (right) and the normalized percentage of how many configurations in that group activated the fibers in each pathway. 100% means that every configuration in the effect group activated that specific fiber, and 0% means that no configuration in that group activated that specific fiber. Nearly every stimulation configuration in the positive effect group activates nearly every fiber in the DTTm while activating minimal Cm-Pf fibers. The no effect group and the negative effect groups had reduced percentages of configuration activation of the DTTm and increasing activation of the Cm-Pf fibers.

Finite element bioelectric field models and multi-compartment neurons simulations were computed for each animal to predict the activation of both the DTTm and Cm-Pf fiber pathways by the stimulation configurations and amplitudes tested during the behavioral experiments. Individual fibers within the two simulated pathways were deemed activated if their membrane potential reached firing threshold when exposed to the stimulation waveform generated by the given configuration and amplitude. The combined lead locations for all three NHPs relative to targeted CL and DTTm fiber pathways are shown in Fig. 5A. All stimulation configurations with anodes and cathodes placed within these locations were split into three groups based upon whether that configuration, according to the LOR, (a) produced clear positive effects on behavioral performance, (b) had no discernible effect at a majority of amplitudes, or (c) reliably produced negative effects on behavioral performance. The percentage of the number of configurations that activated each neuron was computed for each group, generating a group-wise influence on fiber activation (Fig. 5B). A value of 100% means that every configuration in that group activated that modeled neuron, which was observed in nearly every neuron fiber in the DTTm for the positive effect configuration group with minimal activation of Cm-Pf fibers. Each neuron fiber in the DTTm was activated by approximately 50–75% of the configurations of the no effect group, with a portion of the Cm-Pf fiber group activated by more configurations than the positive effect group. Approximately 50% or less of the configurations in the negative effect group activated neurons in the DTTm, and a considerable number of the Cm-Pf fibers were also activated by 50% of the configurations. Nearly every configuration in the positive effect group demonstrated distinct selective activation of the DTTm fiber pathway, which disappeared into non-selective and diffuse activation of both pathways for configurations that produced no effect.

A multivariable linear mixed effects regression model was implemented to determine the influence of central thalamic fiber pathway activation on behavioral performance (Table 1). The percent activation of both the DTTm and Cm-Pf fiber pathways formed the explanatory variables for each experimental stimulation configuration. The response variable was the LOR of behavioral performance change for that stimulation period. The label of positive effect, no effect, or negative effect was not included in this analysis. Instead, the mixed effects model was clustered by each unique electrode configuration to control for repeated measurements. Activation of the DTTm pathway was shown to significantly improve behavioral performance (β = 0.037, 95% CI [0.0018 to 0.055], *p* < 0.001), and activation of the Cm-Pf pathway was shown to significantly decrease behavioral performance (β = − 0.024, 95% CI [− 0.046 to − 0.001], *p* = 0.039).

**Table 1:** Multivariable mixed effects regression model testing differences in behavioral performance over predicted activation of DTTm and Cm-Pf fibers.

	β	95% CI	*P*-value
DTTm	0.037	[0.018, 0.055]	< 0.001
Cm-Pf fibers	− 0.024	[− 0.046, − 0.001]	0.039

**Figure 6:**
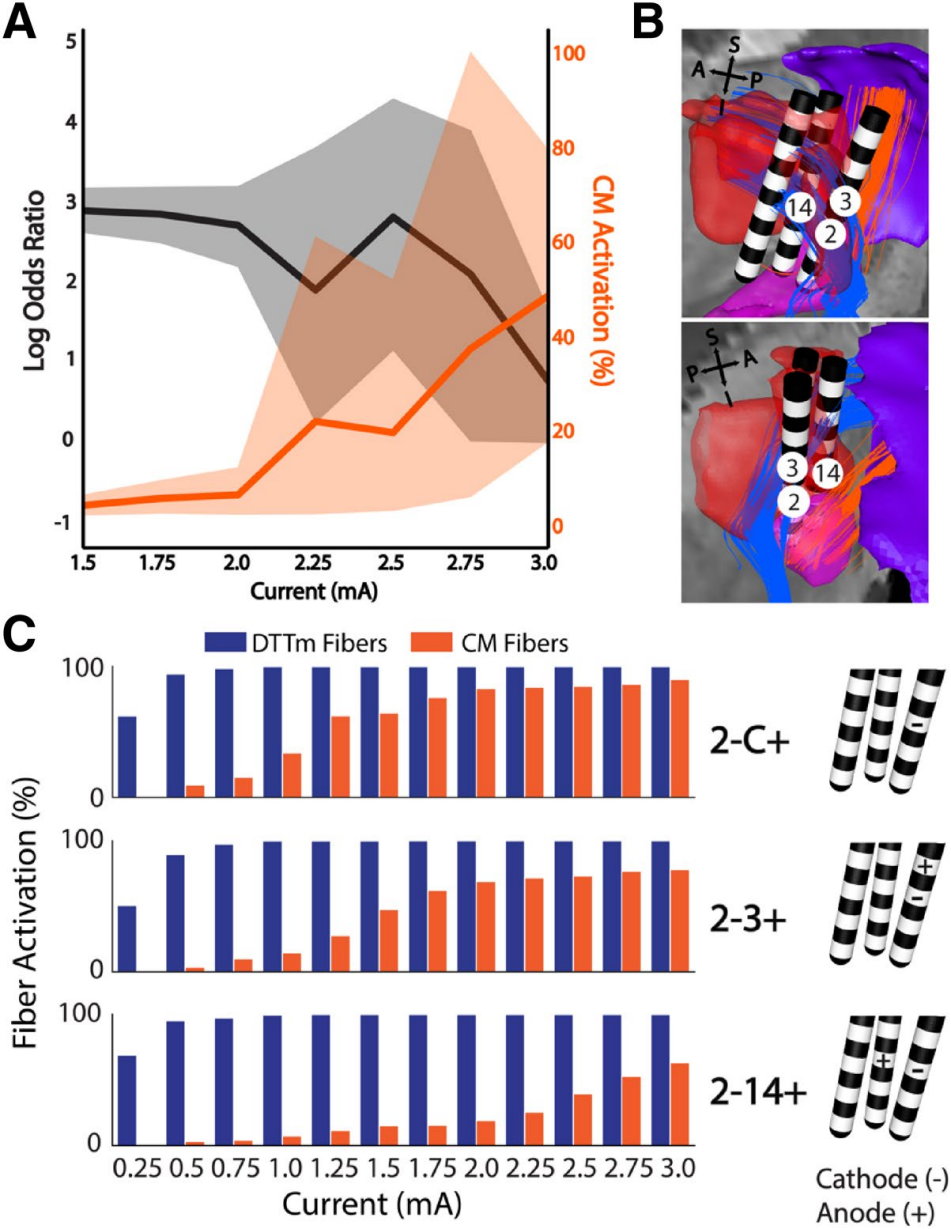
Selective activation of targeted fibers through electrical field-shaping. (**A**) Anode–cathode configurations that resulted in positive behavioral effects show decreased log odds ratios as the current exceeds 2 mA. This decline in performance coincides with increased Cm-Pf fiber activation. **(B)** Lead locations and numbering scheme of the active contacts for NHP3 positioned within the DTTm target (blue) and anteromedially to the Cm-Pf fibers (orange). **(C)** Fiber activation profiles for the DTTm (blue) and Cm-Pf fibers (orange) for three configurations in NHP 3 in which the standard monopolar (2-C +) and bipolar (2–3 +) each show early activation of Cm-Pf fibers. These two configurations were not effective in facilitating behavioral performance despite the cathodic contact being in prime position to activate the DTTm. The third configuration, which utilizes field shaping across multiple leads (2–14 +), was one of the most effective configurations because it provided a larger working range of amplitudes that activated the target DTTm before spreading into the Cm-Pf pathway.

Performance during stimulation was best at lower amplitudes for these configurations and then tapered to zero as stimulation was increased to 3 mA (Fig. 6A, black line), which demonstrated a clear window of optimal effect. For configurations in the positive effect group, the Cm-Pf pathway around 2 mA reaches approximately 50% activation at 3 mA (Fig. 6A, orange line). The configurations in both animals that produced the largest effect on performance utilized multi-lead field shaping with cathodes on the caudal lead and anodes on the rostral lead. The shape of these configurations is more aligned with the DTTm projection through the CL nucleus. Typical cathodic, monopolar stimulation, and bipolar stimulation (within a single lead) did not produce the same effects, and most monopolar stimulations produced stimulation-induced side effects or decreased performance. Configurations utilizing other contacts either resulted in no effect compared to off stimulation or drastically decreased behavioral performance when stimulation was applied.

The numbering scheme and placement of the leads relative to the fiber pathways in NHP3 are shown in Fig. 6B. Contacts 2 and 3 are located on the caudal lead and contact 14 is located on the rostral and more lateral lead. The caudal lead was positioned both where the DTTm enters the CL nucleus and in close proximity to the Cm-Pf complex and fibers. Cathodic stimulation of contact 2 (2-C+) is well positioned to activate the DTTm pathway; however, the generated field spreads uniformly in all directions, activating the Cm-Pf pathway at low amplitudes (Fig. 6C, top). A typical bipolar configuration, with a single lead (2–3 +) creates a directional stimulation field, is able to maintain DTTm activation while reducing Cm-Pf activation. A marked reduction of Cm-Pf activation was demonstrated by shaping the stimulation field across the two leads (2–14+), which orients current both along the direction of the DTTm fibers and orthogonal to the Cm-Pf fibers. This configuration provided the largest window of DTTm activation while minimizing activation of Cm-Pf. As shown in Fig. 6A, there is still an amplitude limit for the optimal configurations and a rollover of the positive effects as performance is suppressed (see Supplementary Fig. 2 for examples). In summary these results highlight the sensitivity between facilitation and suppression of behavioral performance based upon minor variations in the location and shape of the stimulation field relative to the central thalamic fiber pathways.

## Discussion

We developed a biophysical modeling framework^49^ to study DBS within the NHP thalamus to identify the central thalamic pathways associated with significant changes in behavioral performance. Our results demonstrate that selective activation of the DTTm fiber pathway that projects through the CL nucleus, and not the Cm-Pf complex fiber projections, facilitates performance. Although we did this study in healthy macaques, the identified stimulation target can be directly translated into therapeutic options for patients suffering from impaired arousal regulation and enduring cognitive dysfunction^8,47^. In a prior study^35^, we found that shaping the DBS electrical field within the ‘wing’ of CL resulted in robust behavioral facilitation and enhancement of frontal and striatal population activity. These findings are consistent with the behavioral and physiological effects of conventional CT-DBS in a case study in a very severe TBI patient^10^. These new results disaggregate the central thalamus by isolating contributions from CL and DTTm from the contributions of the Cm-Pf complex (Fig. 5B). We hypothesize that two mechanisms may explain these behavioral results: (1) an intrathalamic inhibitory network similar to that defined in the rodent^43,44^, (2) the distinct roles the two pathways play in controlling the anterior forebrain mesocircuit^54^, a system involving the thalamus, frontal cortex, and basal ganglia that regulates the overall level of activity in the anterior forebrain. While this two-source hypothesis needs additional testing, the findings presented here provide a rigorous methodology for optimizing the position of segmented single leads and multi-lead systems to selectively target the cell bodies of CL and the DTTm pathway and to avoid the fiber projections emanating from Cm-Pf. The present results demonstrate that isolated activation of the DTTm pathway projecting from CL to frontostriatal targets facilitates behavioral performance. In contrast, mixed activation of the DTTm and fibers projecting from the Cm-Pf complex through the TRN either interrupts or mitigates these facilitation effects and invites further interpretation. Such observations may seem counterintuitive as both CL^10,22–24,35^ and Cm-Pf^55^ activation with DBS have been reported in awake humans and animals as facilitating arousal and behavior. The anatomical and physiological specializations of CL and Cm-Pf afferents, the postsynaptic effects of thalamic stimulation in the striatum, and the possible role of intrathalamic inhibition mediated through the TRN suggest, however, that marked differences in the impact of DBS should be anticipated. We discuss these considerations below.

Although both CL and Cm-Pf have strong striatal projections, their patterns of innervations within the striatum are markedly different, both regionally and with respect to cellular elements and cell types innervated. Single fiber studies note that CL afferents make en passant synapses in TRN before fanning out broadly over the rostral striatum^56^; by contrast, Cm-Pf fibers project heavily into regionally precise zones of the striatum and form bushy local arborizations^41,53,57,58^. CL and Pf afferents are known to project into the main neuronal populations of the striatum, the medium spiny neurons^59,60^, whereas Cm neurons synapse on the local cholinergic inhibitory neurons^53^. Most importantly, CL fibers have strong and broad frontostriatal projections that strongly activate the entire frontal/prefrontal cortex and rostral striatum with high-frequency stimulation^26,32,35^.

Despite these distinctions, improved arousal and facilitation of behavior have been reported for electrical stimulation of both CL and Cm-Pf. In rodent studies, electrical stimulation of CL facilitates object recognition memory^22^, working memory^25^ and decision-making^23,24^. In healthy NHPs, CL dominant stimulation, that includes the DTTm as shown here, facilitates sustained attention, working memory, and pattern-recognition behaviors^35^. In humans, CL stimulation has shown facilitation of a range of cognitive behaviors including motor executive function and speech production^10^. However, human studies also report speech facilitation with Cm-Pf stimulation^55^ and restoration of arousal in severe brain injury^15,16^. Given evidence that activation of either CL or Cm-Pf might produce behavioral facilitation, how can the findings in Fig. 5 be reconciled?

In rodents, Crabtree and Issac^43^ demonstrated a structural basis for a rich system of intra-thalamic inhibitory interactions and characterized two important findings relevant to the present results: (1) a rich network exists for local inhibition within the thalamus of separate sensory nuclei or motor nuclei; these inhibitory networks appear to be local to either sensory or motor thalamic nuclei; and (2) a cross sensory-to-motor thalamus pathway via the inhibition of the anterior intralaminar group by the caudal intralaminar group. Activation of the caudal intralaminar group produced powerful inhibition and suppression of neuronal firing in the anterior group via a disynaptic connection with TRN. These findings suggest an important motif of intra-thalamic inhibition of the two intralaminar nuclear groups in the thalamus. However, an important distinction in the rodent compared with feline or primate thalamus is the inclusion by Crabtree and Issac of CL as part of the caudal intralaminar group, in large part because the Cm-Pf nucleus is not present in the rodent^37^. In comparison, Cm-Pf in primates is massively expanded^12,37^, and CL has been classified as a component of the rostral intralaminar group^37^. Jones^37^ particularly notes that the paralamellar MD densocellular components can be considered posterior cells of the CL nucleus; these neurons strongly project to frontal and pre-frontal cortices and are contiguous with medial aspects of Cm-Pf and the anterior aspects of Pf. Several anatomists have argued for these regions to be included in the human CL nucleus^37^. Detailed studies of Cm-Pf and CL interactions through the TRN are not available in non-human primate, and our modeling can be guided only by the observations in the rodent. A direct inhibitory effect on CL and surrounding association nuclei through TRN projections activated by the Cm-Pf-TRN fiber bundle could explain the apparent interference when activation is balanced in the DTTm and Cm-Pf-TRN fibers and the mitigation of this interference, with a ‘push–pull’ effect tipping toward behavioral release as the DTTm becomes relatively more engaged (Fig. 5).

### DTTm activation facilitates selective activation of frontostriatal neurons in the awake state

Prior studies have demonstrated that facilitation of cognitively mediated behaviors in the healthy NHP requires a sufficiently powerful activation of frontal and striatal neurons to alter local field potential^35^ and individual neuronal spiking dynamics (Baker et al.^61^). In the awake state, both frontal neocortical neurons and striatal medium spiny neurons are depolarized and receive a high rate of synaptic input^62,63^. Thus, to create sufficient impact as to be measurable in behavioral facilitation, the effects of DBS must be both spatially broad and strongly effective across frontostriatal populations. Stimulation of CL with microelectrode techniques in awake NHPs demonstrated modest effects of behavioral facilitation^53^. In contrast, the marked increase of behavioral facilitation achieved by effective geometries produced by ‘field-shaping’ within the central thalamus (fsCT-DBS) when directly compared with conventional CT-DBS, can be first understood in the context of bulk activation across frontostriatal networks^35^. In human subjects, bulk activation of frontostriatal neuronal populations has been demonstrated as a common mechanism underlying a variety of effective pharmacological^45,64^ and electrophysiological stimulation^65^ treatment methods aimed at improving arousal regulation in the injured brain.

In rodents, optogenetic stimulation of local neuronal populations within the central thalamus demonstrates that CL stimulation uniquely activates the entire frontostriatal system as measured at the whole brain level using functional magnetic resonance imaging^32^. The selective effect of stimulation of DTTm fibers demonstrated here (Fig. 5) is consistent with CL stimulation providing a broad excitatory input across frontal cortical and striatal regions. That even limited co-activation of the Cm-Pf- > TRN fibers had a suppressive effect on behavior draws attention to the further distinctions of CL neurons and those within the parafascularis (Pf) and centromedian (Cm) nuclei.

The distinctions between CL and Cm-Pf neurons extend to their postsynaptic effects on inhibitory medium spiny neurons (MSNs), the neurons that project out of the striatum to the globus pallidus (internal division). Whole-cell patch-clamp studies of MSNs optogenetically activated by either CL or Pf afferents show that CL afferents act through AMPA receptors and are more effective in driving MSN action potentials. Additionally, the Pf afferents, which act via NMDA receptors, generate long-term depression through mechanisms of synaptic plasticity^60^. These physiological distinctions likely provide additional contributions to the mitigation of behavioral facilitation achieved through DTTm activation when Cm-Pf fibers are co-activated because these projections continue in the striatum to MSNs. We hypothesize that the excitation of MSNs by CL leads to disynaptic disinhibition of the thalamus through the anterior forebrain mesocircuit^45,54^, and that co-activation of Pf fibers can oppose this thalamic disinhibition through suppression of the MSNs. Thus, the balance between CL-DTTm and CM-Pf afferents to the MSNs becomes a means by which the overall activity level of the thalamus can be regulated.

Important distinctions at the cortical level are also expected to influence the impact of CL versus Cm-Pf activations; whereas CL innervates the cortex broadly, Cm-Pf projections are comparatively sparse^37^. Within the neocortex, the broad innervation of supragranular and infragranular layers by CL afferents is associated with supralinear summation of effects across cortical columns^66^. Collectively, it is likely that the encroachment of activation on Cm-Pf reduces the bulk activation of frontal cortical and striatal regions through local synaptic effects within the striatum where short-term depression may affect patchy regions of striatum innervated by Cm-Pf projections and interfere with behavioral facilitation^53,60^. Additionally, powerful inhibition of cell bodies within parts of CL or paralaminar thalamic regions (that contain neurons with identical properties^37,67^ via feedback inhibition from the TRN^43^ as described above may suppress thalamic output not captured by direct electrical stimulation.

In comparison to the broad bulk activation required to produce behavioral facilitation with CT-DBS in DTTm, recent work in anesthetized NHPs has demonstrated that very local stimulation within the CL nucleus using multiple 25um contacts spaced 200um apart could produce arousal from propofol and isoflurane anesthesia^33^. The effective electrotonic length of these microprobe contacts, which determines the current flow achieved locally^68^, is very short compared to the broad region activated by the fsCT-DBS configurations studied here. Of note, in this study stimulation at 50 Hz but not 200 Hz was effective in producing arousal during anesthesia. In comparison, in the awake monkeys studied here, stimulation at 150–225 Hz demonstrated the strongest behavioral facilitation and robust activation in frontal and striatal regions, as reflected by a marked increase in the beta and gamma frequency range and a decrease in the lower frequency bands measured directly in these locations^35^. These differences likely reflect the need, in addition to achieving broad activation in the awake state, to increase levels of background synaptic activity received by neocortical and striatal neurons past particular thresholds^69–71^. Intrinsic integrative properties of individual neocortical neurons change with increasing levels of background synaptic input^72^. In order to trigger dendritic electrogenesis in neocortical neurons, across all layers, incoming excitatory inputs must have frequencies higher than ~ 130 Hz^69–71^. Similarly, the primary output neurons of the striatum, medium spiny neurons, require very high rates of background synaptic inputs to maintain membrane depolarization sufficient to generate action potentials^73^. Both mechanisms likely play a role in the requirement for high-frequency stimulation in the awake state^74^. The selective effect of 50 Hz CL stimulation in the anesthetized monkey may alternatively reflect antidromic activation of brainstem cholinergic and/or noradrenergic fibers that innervate CL. The brainstem neurons projecting to CL are known to have resonant properties at ~ 40–50 Hz whereas higher frequencies of stimulation actually block action potentials^75,76^, perhaps accounting for why others saw no effect during high-frequency stimulation^33^. In comparison, a similar study^34^ used high frequency CT-DBS in anesthetized NHPs and demonstrated clear arousal effects and resumption of behavioral performance that corresponded with marked changes in large-scale cortical activity patterns. Importantly, another group recently developed a novel whole brain fMRI approach in healthy, anesthetized NHPs^77^ to explore thalamic DBS in order to restore consciousness during anesthesia^78^. These three unique studies are concordant with our approach here in healthy behaving NHPs.

### Clinical implications

The effects shown here provide important insights into ongoing^47^ and future clinical trials using CT-DBS as an investigational therapy to restore cognitive functions within the broad spectrum of SBI patients ranging from very poor recovery (GOSE 3–4) to moderate/lower good recovery but with persistent fatigue and cognitive dysfunction (GOSE 5–7). More broadly, the general approach developed here could assist in the planning of DBS lead placement in other investigational targets to treat various neurological and neuropsychiatric disorders. For example, in treatment-resistant depression (TRD), DBS within the subcallosal cingulate white matter has demonstrated immediate and enduring effects in patients^79^. However, the ‘optimal’ stimulation target for this specific disease is still under investigation^80,81^ and outcomes have been heterogeneous across the various teams investigating DBS for TRD (reviewed in^82^). Accumulating evidence suggests that even within established DBS targets for Parkinson’s disease, such as the subthalamic nucleus (STN) and Globus pallidus interna (GPi), there are circumscribed regions where therapeutic DBS can be ‘optimized’ to limit activation of adjacent regions that result in consistent adverse ‘off-target’ side effects^83,84^. In addition, this computational modeling framework could help in the interpretation of variable outcomes and potentially lead to better and more consistent clinical outcomes as new DBS targets are investigated.

### Future directions

In this study we focused on comparing DTTm fiber and Cm-Pf fiber activations during CT-DBS induced relative changes in performance. Additional fibers originating from adjacent nuclei like medial dorsalis (MD) or *en passant* fibers passing near the CL and Cm-Pf nuclei may also play a role but were not modeled. A comprehensive and exhaustive comparison of all fiber bundles was limited by the derived nature of the modeled fibers and a consequent multiple comparisons problem presented by the number of animals and limited number of experimental data points. Future studies could more systematically examine the possible contribution of nearby pathways to behavioral performance by consistently targeting those pathways across multiple subjects. These investigations could include fibers from MD projecting to medial and orbitofrontal cortex or the fasciculus retroflexus, a prominent fiber pathway traversing the posterior region of Pf (studies in NHP2 suggest strong behavioral suppression with selective activation of the fasciculus retroflexus pathway^85^). Finally, the possible contribution of intra-thalamic connections between Cm-Pf and the ventral anterior and ventral lateral nuclei, which are known to produce motor impairments when lesioned^86^, could be systematically studied.

## Methods

### Animal care

The methods reported follow the ARRIVE guidelines (https://arriveguidelines.org/). All work was performed in strict accordance with the National Institutes of Health Guidelines for Use of Animals in research and under an approved protocol from the Weill Cornell Medical College Institutional Animal Care and Use Committee (IACUC). Animals were cared for by the Research Animal Resource Center (RARC) at Weill Cornell Medicine.

### Imaging and surgical procedure

A detailed description of the imaging and surgical procedures can be found in prior publications^35,87^. Briefly, following successful behavioral training each animal was anesthetized and placed in a magnetic resonance (MR) compatible stereotaxic surgical frame (Kopf Instruments, 1430 M) for presurgical MR (3 T Siemens) and computed tomography (Siemens PET/CT) imaging. These images were then registered to a high-resolution MRI-DTI macaque full-brain atlas with 241 segmented anatomical structures (http://www.civm.duhs.duke.edu/rhesusatlas/)^50^ for surgical planning using 3D Slicer (https://www.slicer.org/). Segmentations of the thalamic nuclei, cortical regions and DBS leads were used in the modeling software to plan the entry and end points for each DBS lead and the custom cephalic chambers (Gray Matter Research, LLC). Contrast enhanced MR imaging (Ablavar, Lantheus Medical Imaging Inc., North Bellerica, MA) was used to visualize the vasculature and plan the trans-ventricular DBS lead trajectories that typically had an entry point at the somatosensory cortex and end point within the Cm-Pf nucleus. Following insertion, the DBS leads were secured and housed within a Deep Brain Recording and Stimulation (DBRS) device (Gray Matter Research, LLC) customized for each animal. In addition to the DBRS system, a head fixation post (Gray Matter Research, LLC), Titanium grounding plates and grounding screws, and a 32–128 microelectrode microdrive was implanted in each animal using standard sterile surgical techniques^35,87^. Electrophysiological signals were recorded in each animal but not analyzed in this study. The animals were allowed to recover for 30–45 days before resuming behavioral training and the DBS experiments. A computed tomography (CT) scan was performed 30 days following implantation to visualize the metal artifacts created by each DBS lead contact and was used reconstruct the DBS lead locations relative to the thalamic nuclei in the biophysical model.

### Subjects

Three adult male (11, 10 and 12 kg) non-human primates (NHP), *Macaca mulatta*, were used in this study. We combined previously collected data collected from two animals^35^, NHP1 and NHP2, with new data collected in a third animal (NHP3) performing a visuomotor reaction time task, as detailed below. All subjects were healthy and each acted as a control, whereby many CT-DBS parameters and repeated experiments produced no significant effects on behavioral performance.

### Behavioral tasks

Two animals (NHP1 and NHP2) were trained to continuously perform a variable delay period visuomotor reaction-time task “S1–S2,” or “phasic alerting” paradigm used in both human and NHP studies^53,88–91^. Briefly, the structure of this task is initiated by the appearance of a target (a black/red checkerboard or dartboard 5-degree × 5-degree of visual angle) at 1 of 9 locations (chosen at random on each trial) on a CRT monitor positioned in front of the animal. After a 1-s period of stable fixation of the target, the target underwent contrast reversal at 10 Hz for a variable delay period until changing to a black/green checkerboard or dartboard. The transition to black/green from black/red was the ‘GO’ signal for the animal to contact the infrared (IR) touch switch located within the primate chair (Crist Instrument Co. Inc., Hagerstown, MD). The variable delay period was randomly drawn from a normal distribution with a mean of 2500 ms and a standard deviation of 250 ms. A trial was considered to be incorrect if the NHP broke fixation prior to the ‘GO’ cue or touched the IR switch before or within 250 ms after the ‘GO’ cue or failed to respond within 800 ms after the green target. The third animal, NHP3, was trained to perform a similar variable delay period visuomotor reaction time task, but this task required the animal to initiate each trial of the task by placing its hand over the IR switch, i.e. to engage the task. The IR touch switch would initiate the task in a customized program (NIMH MonkeyLogic^92^) that controlled the visual appearance of a central grayscale Gabor (3-degrees of visual angle) embedded in a 1/f noise pattern that filled the LCD monitor. The animal had to maintain fixation on the Gabor (a circular window with 5–8° of visual angle in extent) as the spatial phase changed (every 300 ms) for a variable number of phase transitions (lasting 900–3000 s) before changing orientation and phase (from 0 to 22.5 or 45°). The change in orientation acted as the ‘GO’ signal and the animal had to remove its hand from the IR switch within 900 ms to receive a juice reward. Any given trial was considered to be ‘engaged’ when the IR switch was triggered following a correct or incorrect trial, regardless of the outcome. For example, the animal might touch the switch, which initiated the task sequence and then it would break fixation of the Gabor, thus aborting the trial; or it might maintain fixation and remove its hand from the IR switch shortly after triggering the task sequence. Although different visually and in motor sequence, the two tasks require sustained attention and stable fixation over variable delay periods and over extended periods of time, typically lasting 1.5–3 h.

### Central thalamic deep brain stimulation

The DBS leads were scaled for the NHP^48^ based on the relative dimensions of a commercial DBS lead (Medtronic 3387 lead, also see the 3389 lead). In this study the DBS leads (0.84 mm OD) had six platinum/iridium annular contacts, each 0.5 mm in height, with an intra-lead spacing of 0.5 mm and insulated by polyurethane (NuMED, Inc. Hopkinton, NY). A four-channel Multi Channel Systems MCS GmbH stimulator (STG4004-3.2 mA) with a compliance of 120 V was connected to the DBS leads to provide independent current controlled stimulation. The stimulation waveform consisted of an 80 µs square cathodal pulse followed by an isoelectric period of 60 µs and ended with a 400 µs square anodal pulse to balance the total 5:1 cathode to anode charge. Each pulse lasted a total of 540us. This stimulation waveform mirrors the output of the Medtronic Inc. clinical system (as described in^93^) and provided safe and preferential activation of large myelinated axons^94,95^. In this study stimulation frequencies of 150, 175, 200, and 225 Hz, and amplitudes 0.25–3.0 mA were used. Following implantation, a monopolar review (150, or 200 Hz, ramp from 0.25 to 3 mA) for each contact was conducted to identify any adverse off-target effects of stimulation. For example, consistent paresthesia’s, arm, leg, body or eye movements. A wide range of intra-lead and inter-lead anode–cathode configurations were explored during the experimental sessions in each animal. In this study configurations that led to behavioral facilitation were then used in the model. In NHP1 10 configurations, 462 DBS periods, and 42 experimental sessions were included. In NHP2 4 configurations, 11 of DBS periods during one experimental sessions were included. In NHP3 25 configurations, 116 DBS periods and 15 experimental sessions were included. CT-DBS was delivered in a unblinded fashion by using multiple trial blocks of continuous stimulation for a variable number of trials (25–100 trials).

### Computational modeling of electrical stimulation in the thalamus (R1-C3, R1-C4, R2-C2, R2-C4, R2-C6)

In our previous study^35^, we used a simple model of axonal node activations using a diffusion tensor brain template of the NHP^96^ in combination with subject-specific imaging and histological reconstruction of the DBS lead locations. Here, we used an animal-specific virtual DBS pre-surgical planning model^49^ was used to set the lead locations and inter-lead spacing in NHP3 to optimize targeting of the ‘wing’ of the central lateral (CL) nucleus^11^ which contains the highest concentration of DTTm fibers^36^. Following implantation of the DBS leads, a 30-day postoperative computed tomography scan was acquired to identify the DBS lead locations based on the imaging artifacts produced by the lead contacts (see insets in Fig. 2A). The postoperative CT scan was then rigidly registered to the preoperative surgical imaging to identify the individual contacts of the DBS leads relative to the thalamic targets. The DBS lead locations in NHP1 and NHP2 were derived from imaging and later confirmed using standard post-mortem histology techniques^35^ and in NHP3, the lead locations were derived from CT imaging alone. Anatomical nuclei were identified based upon nonlinear registration of the preoperative MRI to a high-resolution macaque atlas^50^ using symmetric normalization^97^. Diffusion imaging was not acquired in NHP1 or NHP2 prior to implantation. Since healthy, adult NHPs were used in this study, a high-resolution diffusion MRI dataset, acquired ex vivo from one adult male *Macaca mulatta* (see Sani et al. 2019 for acquisition and pre-processing details), was nonlinearly registered to the Calabrese atlas for deterministic tractography using DSI Studio (http://dsi-studio.labsolver.org). The in-plane resolution was 0.250 mm, and the slice thickness was 0.254 mm. The high-resolution macaque atlas and high-resolution ex-vivo diffusion dataset offered significant advantages to our stimulation models compared to acquisition of lower quality individual diffusion scans from NHP3. The diffusion data were reconstructed using generalized q-sampling imaging^98^ with a diffusion sampling length ratio of 1.5. Three fiber orientations per voxel were resolved with an eightfold orientation distribution function (ODF) tessellation. The two reconstructed tracts in the central thalamus were: the DTTm, seeded in the pedunculopontine nucleus with CL and prefrontal cortex as regions of interest (ROIs); Cm-Pf fibers (seeded in the Cm-Pf nucleus with TRN as the ROI).

The finite element method (FEM) was used in SCIRun 5.0 (SCI Institute, University of Utah, Salt Lake City, UT, http://sci.utah.edu/software/scirun.html) to solve the bioelectric field problem and compute the voltage distribution through simulated brain tissue surrounding the DBS leads. Similar computational models used to predict the effects of DBS have been validated in prior human and NHP studies^99,100^. A FEM tetrahedral mesh was created for each animal, and the DBS leads were positioned in the model based upon the both the postoperative computed tomography artifact (NHP3 only) and histology for NHP1 and NHP2 (see^35^). Isotropic conductivities were applied for the DBS contacts at sigma = 1 × 10^6 ^S m^−1^, the non-conductive shaft segments at sigma = 1 × 10^–10 ^S m^−1^, and brain tissue at sigma = 0.2 S m^−1^. The conductance of the 100um thick encapsulation layer between the DBS and brain tissue was adjusted to match the average measured impedance in vitro for each animal. The bioelectric field forward problem was solved using a current point source, set to − 1 mA, at the center of the active contact. The outer surface of the FEM was set as the distant return for monopolar simulations.

A multi-compartment neuron model was placed along each tract for the two reconstructed fiber bundles. The neurons were modeled as 2 µm myelinated axons with a 323.2 µm spacing between nodes of Ranvier and biophysical properties derived from the MRG model^101^. The computed electric potentials from each FEM model were linearly interpolated onto the neuron models, and NEURON 7.4 was used to simulate the neuronal response to the extracellular DBS waveform. Each neuron was determined to be activated if a compartment reached firing threshold in response to the applied waveform at a given amplitude. The percentage of activated neurons was computed for each fiber bundle. Traditional methods of computing the total volume of tissue activated were not used due to the complex multi-polar, cross-lead stimulation configurations performed in this study.

### Statistical analysis

To provide a visual estimation of the animal’s performance across experimental sessions the series of correct, ‘1’ and incorrect, ‘0’ trials were used to generate a state space model^53^. This smooth estimate of percentile performance, from 0 to 100% was used to visualize task performance and task engagement as a function of trial number. The odds ratio is the probability of the animal performing a correct trial during DBS divided by the probability of performing a correct trial prior to DBS onset. The log of this ratio is the log odds ratio (LOR). Positive LOR values correspond to a greater probability of the animal performing a correct trial during DBS.

The log of the odds ratio (LOR) was used to quantify the effect size of DBS during a block of trials compared to performance during the block of trials prior to DBS. The LOR was computed as the log of the ratio of the odds of correctly performing a trial during a DBS ON period to the odds of correctly performing a trial during the immediately prior DBS OFF period. Odds ratios for all DBS periods were computed and subjected to a 95% confidence based on the standard error and the total number of trials in both the ON and OFF periods. A minimum of 20 trials prior to the onset and 20 trials during DBS were required for a DBS period to be included in this study. Statistical significance was determined at an α level of *p* < 0.05.

To assess whether behavioral performance changed based upon activation of specific central thalamic pathways, a multivariable mixed effects regression model was implemented. The percent activation of both the DTTm and Cm-Pf fiber pathways was calculated for each DBS period based upon the current configuration and amplitude. The percent activation of each fiber bundle formed the explanatory variables to determine if stimulation of these pathways could predict the experimentally observed change in performance measured as the LOR. A total of 589 DBS periods and 39 unique stimulation configurations across the three animals were included in this analysis. Random intercepts were included to allow for correlation among repeat outcome measures within stimulation configurations and random effects for each subject.

## Supplementary Information

Supplementary Information.
