# Supplementary material for: Selective activation of central thalamic fiber pathway facilitates behavioral performance in healthy non-human primates
Source: Sci Rep. 2021 Nov 29;11:23054. doi: 10.1038/s41598-021-02270-7 (PMC8630225; doi:10.1038/s41598-021-02270-7)
Supplement: Supplementary file 1 — Supplementary Information. [file 41598_2021_2270_MOESM1_ESM.docx]

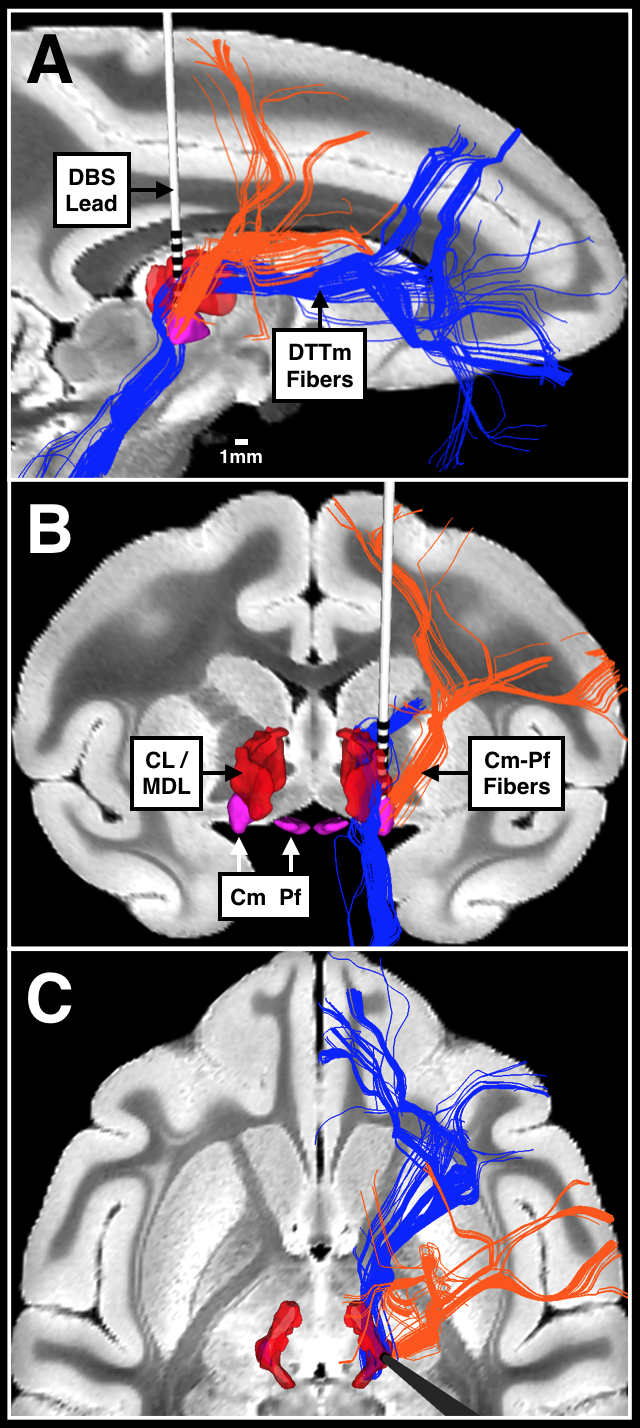


**Supplementary Figure 1. Targeting the central lateral thalamic nucleus and associated fiber pathways in a healthy NHP with scaled DBS leads (A)** Sagittal view of the presurgical implant trajectory for one of the three 6-contact DBS leads chronically implanted into the right thalamus of NHP3. The targeted fiber pathway of the DTTm, shown in blue, illustrates the trajectory of brainstem and anterior forebrain passage of fibers through and/or originating in the CL-MDL nuclei, shown in red. **(B)** Coronal view of the presurgical implant trajectory. The CL-MDL nuclei are shown in red, Cm and Pf nuclei are shown in magenta, and the associated Cm-Pf fibers are shown in orange. **(C)** Axial view of the presurgical implant trajectory. Note the prominent anterior projections of the DTTm fibers (blue) into the prefrontal cortex as compared to the more lateral projections of the Cm-Pf fibers (orange).


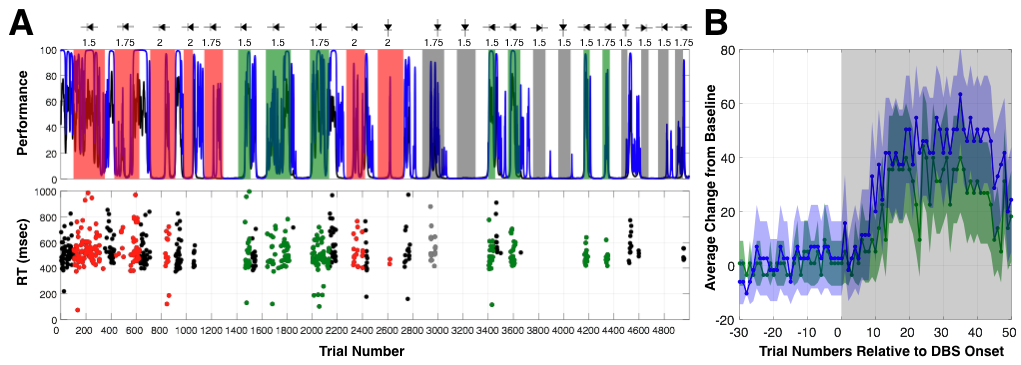


**Supplementary Figure 2. The effects of CT-DBS amplitude and configuration on engagement and performance in NHP3 (A)** The performance estimate of NHP3 on the task is shown in the upper plot as a smoothly varying black line and the animals’ engagement (see Materials and Methods) across trials is shown with a blue line (*53*). Periods of continuous high-frequency fsCT-DBS are colored according to the significance of the LOR value (p<0.05); facilitation in dark green, suppression in red, and gray for no effect. Stimulation amplitudes are noted above each CT-DBS period. The diode symbol above each CT-DBS period indicates the anode-cathode configuration used:
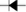
 corresponds to cathodes set on contacts 1 and 2 (DBS lead 1) and anodes set on contacts 14 and 15 (DBS lead 3),
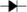
 corresponds to anodes set on contacts 1 and 2 (DBS lead 1) and cathodes set on contacts 14 and 15 (DBS lead 3),
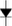
 corresponds to anodes set on contact 1 (DBS lead 1) and contact 14 (DBS lead 3) and cathodes set on contact 2 (DBS lead 1) and contact 15 (DBS lead 3). The lower plot shows the reaction times of correctly performed trials with the same color code; black during the CT-DBS OFF periods. **(B)** Average change in behavioral performance (dark green) and engagement in the task (blue) when field-shaping CT-DBS (23 periods) was used in NHP3 and resulted in facilitation of performance. Each curve is normalized to pre-stimulation performance levels, including ±95% CI. Here CT-DBS periods included configurations where one or two cathodes were set on contacts 1, 2, and/or 3 (DBS lead 1) and one or two anodes were set on contacts 13, 14, and/or 15 (DBS lead 3), as illustrated in Figure 5C.
